# A Multicenter, Randomized, Open-Labeled, Parallel Group Trial of Sildenafil in Alcohol-Associated Erectile Dysfunction: The Impact on Psychosocial Outcomes

**DOI:** 10.3390/ijerph6092510

**Published:** 2009-09-23

**Authors:** Alexander M. Ponizovsky, Lev Averbuch, Ira Radomislensky, Alexander Grinshpoon

**Affiliations:** 1 Mental Health Services, Ministry of Health, 2 Ben Tabai St., Jerusalem 93591, Israel; E-Mail:pirsumus@inter.net.il; 2 The Department of Substance Dependence Treatment, Ministry of Health, 20 King David St., Jerusalem 91010, Israel; E-Mail:lev00@walla.com; 3 Tirat Carmel Mental Health Center, Ministry of Health, 17 Ha’ela St., ‘Tirat Carmel 30200, Israel Sha’ar Menashe Mental Health Center, Ministry of Health, Mobile Post Hefer 37806, Israel; E-Mail:Alexander.Grinshpoon@sm.health.gov.il

**Keywords:** alcohol dependence, erectile dysfunction, sildenafil, depression, functioning, self-esteem, social support

## Abstract

To examine the effect of sildenafil on erectile dysfunction (ED) and psychosocial outcomes in alcohol-dependent (AD) men, 108 men with these diagnoses were randomly assigned to either take sildenafil (50 mg) as add-on to standard treatment for AD, or the same treatment without sildenafil, for 12 weeks. Only 50 patients in sildenafil group and 51 in control group twice completed the International Index of Erectile Function (IIEF) and a battery of self-report questionnaires. IIEF scores and psychosocial functioning, self-esteem and support from friends improved only for sildenafil-treated patients (P < 0.001). The high effect sizes suggest that the observed benefits are unlikely to be a placebo effect, although their unspecific nature could not be ruled out. In men with ED associated with AD, sildenafil improves both ED and psychosocial outcomes. Further placebo-controlled clinical trial is warranted.

## 1. Introduction

Alcohol dependent men commonly suffer from erectile dysfunction (ED) [1–4], and conversely, men having ED are frequently chronic alcohol addicts [5,6]. Particularly those in remission, who were forcibly withdrawn from alcohol, often complain about impotence and report it as a “cause” for relapse. Findings from previous research show that alcohol increasingly inhibits normal erectile function [7] and it, in turn, may lead to greater alcohol consumption as a “self-treatment” attempt. Thus, the vicious cycle of ED and heavy alcohol consumption is developed.

Sildenafil citrate (VIAGRA^®^) is reportedly an effective and safe medication indicated for the treatment of ED [8,9]. It is a competitive inhibitor of cGMP-specfic phosphodiesterase type 5. The medication amplifies the effect of sexual stimulation by retarding the degradation of this enzyme. Sildenafil was found effective in several subpopulations of men with ED, including those suffering from diabetes [10,11], hypertension [12,13], spinal cord injuries [14–17], multiple sclerosis [18], depression [20–24], post traumatic stress disorder [25], schizophrenia [26,27], those after resection of the prostate or radical prostatectomy [28], renal transplantation [29], dialytic treatment [30], and those aged 65 years and older [31,32]. Efficacy has been maintained for up to one year with no evidence of tolerance [19].

Adverse events associated with sildenafil use have generally been transient and have mild-to-moderate severity. Primarily they are related to the drug’s vasodilator properties and were reported to occur in nearly 10% of patients in clinical trials [33–36]. The most commonly reported adverse events associated with sildenafil use were: headache (16%), flushing (10%), dyspepsia (7%), and nasal congestion (4%). Drug interactions with sildenafil are minimal, yet its use is contraindicated in those receiving nitrates in any form. Regarding the interaction with alcohol, some evidence of the safety of such combination was recently reported. In a double-blind, randomized, crossover study of the effects of sildenafil in doses of 50 mg co-administered with alcohol in 12 healthy subjects, no significant hemodynamic or pharmacokinetic interactions between the two were observed (Pfizer Inc., personal communication, 2002). However, the hemodynamic changes that occurred during sexual intercourse may be magnified by the consumption of alcohol that requires great caution in the co-administration of sildenafil and alcohol.

Findings from several studies support a biopsychosocial formulation of alcoholics’ sexual problems [1,2]. This approach suggests that marital conflict is a major contributing factor to most sexual problems of alcoholics, apart from the direct physical effects of acute and chronic alcohol intake on the elevated rates of impotence. Therefore, taking into consideration the sensitivity of alcoholics to interpersonal relationships, variables closely related to their marital relationships, such as depression symptoms, psychosocial functioning, social support and self-esteem, would be investigated as the additional indicators of the sildenafil treatment efficacy.

In our recent preliminary, uncontrolled, open-label study we have demonstrated the beneficial effects of sildenafil treatment on sexual function, quality of life and emotional distress in 50 alcohol dependent patients [37]. In the present report, in open-label, multicenter, parallel-group design we evaluated the efficacy of sildenafil treatment in the same population in comparison to untreated control group, with regard to depression, psychosocial functioning, social support and self-esteem in addition to its effect on sexual function. We hypothesizes that the beneficial effect of sildenafil citrate on ED in alcohol dependent men is associated also with improvement in these psychosocial outcomes.

## 2. Method

### 2.1. Study Design

This was a multicenter, prospective, parallel group, randomized, open-label, flexible dose study. It was a 12-week trial conducted simultaneously at 11 outpatient medical centers for alcohol abuse treatment across Israel from January 1, 2005 to June 31, 2006. All centers belong to the Israeli Ministry of Health, and are audited by the Department for the Treatment of Addictions. The Ministry of Health institutional review board approved the study protocol, and all patients provided written informed consent prior to participating in the study.

### 2.2. Patients

Similar to our preliminary report [37], male patients were eligible if they met the following inclusion criteria: 1) were between ages 18 and 50 years; 2) had a ICD-10 diagnosis of AD (F10.2); 3) sought treatment with the aim to stop alcohol consumption; 4) completed a detoxification program not later than the one month preceding the study recruitment; 5) had complaints of ED for at least 12 weeks preceding the study; and 6) had a regular female partner for the study duration.

AD was diagnosed according to International Classification of Diseases, Tenth Edition (ICD-10) criteria [38] adopted in Israel since 1994. The alcohol history was described by the following parameters: 1) age at first alcohol consumption, 2) age at first binge, 3) duration of harmful alcohol consumption, 4) number of prior inpatient or outpatient detoxifications, 5) average alcohol intake in last six months (gram alcohol/drinking day), and 6) number of drinking days during last month. The severity of AD was evaluated according to ICD-10 criteria for three categories according to the frequency of drinking (during the previous 6 months) and amount of alcohol intake. These were: 1) continuous drinkers = (almost) daily alcohol consumption without binges; 2) frequent heavy drinkers = frequent alcohol consumption (more than 3 days/week) with frequent intoxication (more than one/week); and 3) episodic drinkers = less frequent, irregular alcohol consumption with longer (>5 days) sober periods, and some binges (less than one/week).

Alcohol-associated sexual dysfunction (AASD) was defined by carefully elaborated DSM-IV criteria for alcohol-induced sexual dysfunction [39], which includes specific items for impaired desire, arousal (ED), orgasm, and sexual pain. Patients had to have substantial impaired sexual function that caused significant distress, defined by at least one of the following criteria: ED as defined by persistent or recurrent inability to attain an adequate erection until completion of sexual activity; inability to have an orgasm, or ejaculatory delay of at least 10 minutes for masturbation or intercourse.

Patients were not enrolled if they had anatomical penile deformities (e.g., Peyronie’s disease), primary or prior diagnosis of a sexual disorder other than AASD, co-morbid serious medical illnesses (hepatic, renal, neurological, cardiovascular, hematological, diabetes mellitus), suicide risk, acute psychosis, severe depression (with psychotic features), organic brain syndromes or current use of other than alcohol psychoactive substances, drugs or therapies, such as benzodiazipines, sedatives, antidepressants, barbiturates, and antipsychotics. The psychiatric diagnostic assessments were made according to ICD-10 criteria.

### 2.3. Study Protocol

**Figure 1.**
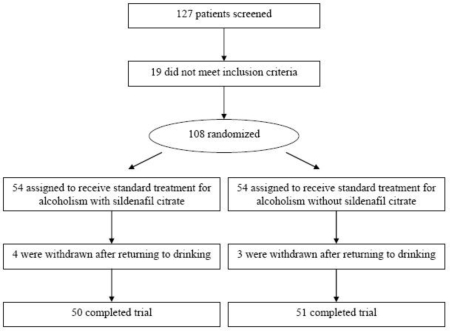
Flow of patients through the trial.

Patients were enrolled during 1.5 years and recruited from outpatient settings and referrals. All patients were evaluated for eligibility at screening (N = 127) (Figure 1) and all consenting patients (N = 108) completed the International Index of Erectile Function (IIEF) [40] to establish their ability for self-evaluation of sexual dysfunction. All patients received a physical examination, including blood pressure, electrocardiogram, and standard biochemistry tests (blood urea nitrogen, uric acid, glucose, total protein, albumin, total bilirubin, total cholesterol, triglycerides, fibrinogen, alkaline phosphatase, SGOT, SGPT, sodium, potassium, chloride, calcium, inorganic phosphorus, bicarbonate, creatinine, and creatine phosphokinase [CPK]) and hematological laboratory tests (hemoglobin, hematocrit, erythrosyte count, white blood cell count [WBC], total and differential WBC count, and platelet count).

Using a random number generator for each incoming participant for treatment decision-making [41], a computer-generated randomization schedule was developed. This resulted in 54 patients assigned to sildenafil citrate as add-on to standard ongoing outpatient program for treatment of AD and another 54 patients assigned to only the standard program without sildenafil. This program involved education and therapy, addressing problems contributing to or resulting from the alcoholism, and learning skills to manage the alcoholism over time. The only restriction to this randomization was that the groups be of equal size. The largest difference in numbers assigned to the two groups at endpoint of the study was 3. Additionally, there were no statistically significant differences between assigned groups at baseline in socio-demographic characteristics (Table 1). At baseline, eligible patients randomly assigned to receive sildenafil were instructed to take one tablet approximately one hour before anticipated sexual activity but not more than once daily. They were also instructed to make at least two attempts at sexual activity weekly. The dose of the drug could be adjusted from 1 to 2 tablets (VIAGRA^®^ was provided by Pfizer Pharmaceuticals Israel Ltd, Herzliya Pituach, Israel). Drug accountability and self-rated and physician-rated assessments were performed at baseline and 12 weeks later. Throughout the study, the investigators monitored and collected any spontaneous reports of adverse events and evaluated their severity and relationship to the study medication.

### 2.4. Outcome Measures

Efficacy was evaluated using a battery of validated measurements. These included the IIEF [40], the Global Life Functioning inventory (GLF) [42], Beck Depression Inventory-short form (BDI-13) [43], General Self-Esteem Scale (RGSES) [44], and the Multidimensional Scale of Perceived Social Support (MSPSS) [45]. Time frame for all measures was the 10 days preceding the assessment.

The IIEF is a self-rated 15-item instrument to assess sexual function in five functional domains: erection, orgasm, desire, intercourse satisfaction and overall satisfaction. Questions are anchored on a 5-point scale with 1 corresponding to “almost never/never” and 5 corresponding to “almost always/always”. A score of 0 means the absence of sexual activity, stimulation or intercourse. The minimum possible total score is 5, and the maximum total is 75. The changes in the IIEF scores quantified the magnitude of the response.

The GLF inventory was developed to tape distress, well-being, functioning and life satisfaction and has been shown to be sensitive to changes and to discriminate well between efficacious treatments [46]. It is based on seven items of the Dupuy’s psychological general wellbeing index to measure wellbeing and distress [47], supplied with six items tapping general functioning and life satisfaction. Each item is rated on a 6-point Likert scale, with higher scores indicating better outcomes. In the present study the GLF was used as a self-evaluation measure of patient general wellbeing and functioning.

The severity of depressive symptoms was assessed using the standard abridged form of the BDI. Each of its 13 categories of symptoms and attitudes scores from 0 (absence of the symptom) to 3 (extreme severity of the symptom). The ranges of total scores are: 0–4, none or minimal, 5–7, mild, 8–15, moderate; and 16 and over, severe depressive symptoms.

The RGSES is a 10-item Likert scale with items answered on a 4-point scale - from “strongly agree” to “strongly disagree”, with higher scores indicating higher self-esteem.

The MSPSS was used as a self-report tool for assessing emotional help and the level of satisfaction with the social support obtained from family, friends and significant others. The scale includes 12 items, each of which refer to the people to whom the respondent would turn if he/she had problems of a personal, health or family nature, as well as financial and employment problems. Responses are scored on a 7-point scale from 1 (“completely disagree”) to 7 (“completely agree”). The MSPSS total score and three subscales scores are computed, with a higher score indicating a greater satisfaction with social support.

For the entire sample (N = 101), internal consistency reliability, as measured by Cronbach’s α coefficient, was satisfactory, specifically: 0.77 for the GLF; 0.80 for the BDI; 0.79 for the RGSES, and 0.91 for the MSPSS.

### 2.5. Statistical Analysis

All analyses were performed with SAS version 9.1 (SAS Institute Inc, Cary, NC, USA). Baseline demographic characteristics were compared using descriptive statistics by χ^2^ and Fisher exact tests (where cell sizes were <5). Mean scores and standard deviations (SDs) were computed and reported. The statistical significance of change from baseline to week 12 (Δ) was evaluated with two-tailed paired t-tests. Effect size is the primary outcome measure, and power for the study was calculated using effect size in each group. Expecting to find differences between the groups of effect size = 0.4 would clarify whether potential observed differences also merit clinical significance. Using a two-group analysis of effect sizes between the sildenafil and control groups, setting alpha at 0.05, power = 0.80 and a two-tailed t-test, a minimum of 52 subjects per group is required. Thus, 52 patients per group met power requirements for these specific aims.

Effect size (ES) for treatment efficacy of erectile dysfunction was calculated as the improvement in IIEF mean score for the sildenafil group minus the improvement in IIEF mean score of the control group over 12 weeks, divided by the standard deviation of the entire sample at baseline. ES for other variables studied was computed using the same equation. Following Cohen’s classic demarcation [48], Middel and associates [49] showed that ES reflects clinical relevance. An ES <0.20 indicates “no change,” an ES ≥0.20 but <0.50 indicates “a small change,” an ES ≥0.50 but <0.80 indicates “a moderate change,” and an ES ≥0.80 indicates “a considerable change”. We also use ES equal 0.5 SD as a universal measure of clinical significance [50].

## 3. Results

A total of 108 men who consented to participate were screened and randomized to sildenafil (n = 54) or standard treatment (n = 54). One hundred-one patients (94.4%; n = 50 for sildenafil and n = 51 for control group) completed all baseline and week 12 endpoint assessments. There were no statistically significant differences between treatment group completers in baseline demographics, except for a greater number of immigrants in the sildenafil-treated group (Table 1). Although the sildenafil-assigned patients did differ significantly from the controls with regard to earlier age at first-time alcohol consumption (17.2 ± 3.4 vs. 21.3 ± 7.0 years; t = 3.75; P < 0.001) and age at first alcohol binge (20.5 ± 6.4 vs. 26.1 ± 8.6 years; t = 3.68, P < 0.001), the groups were similar in the variable of duration of harmful alcohol consumption. Likewise, the groups did not differ significantly in the number of prior inpatient detoxifications, average alcohol intake in the last six months, and the number of drinking days in the month preceding the study. Patients who withdrew (n = 7) were not associated with significantly different baseline demographic characteristics compared with completers.

## Efficacy

### 3.1. Sexual Function

A statistically significant increase in the IIEF mean scores of each sexual function domain was noted among all sildenafil-treated patients (Table 2). In contrast, the mean scores of all the sexual function domains among the control patients decreased over the trial, and in orgasmic function and sexual desire domains this decline was found to be statistically significant (P < 0.05). ES ranged from 0.91 for sexual desire to 1.23 for overall satisfaction domain, and 1.25 for IIEF total score, indicating clinically relevant improvement according Cohen classical demarcation [48]. However, only ESs for sexual desire and overall satisfaction demonstrated clinical importance of the changes using a conservative measure of clinical improvement as 0.5SD [50].

### 3.2. Depression

Baseline levels of depression were generally unrelated to efficacy or treatment satisfaction. Both groups demonstrated a statistically significant reduction in BDI symptom severity scores, with the ES = 0.47 (Table 3).

### 3.3. Psychosocial Functioning

We observed a significant improvement in the GLF total scores as well as in the well-being and functioning subscale mean scores only in sildenafil-treated patients (all P < 0.001), but not in the control group. Correspondingly, ES values ranged from .44 for wellbeing to 0.57 for functioning domains, and 0.63 for GLF total score, reflecting clinically important changes (all > 0.5DS).

### 3.4. Self-Esteem

In parallel to a meaningful depression reduction, the sense of self-esteem improved substantially among individuals of the sildenafil group (P < 0.001), while this remained at pretreatment levels among the controls (ES = 0.61).

### 3.5. Social Support

Similar to self-esteem, from baseline to endpoint a significant improvement was observed in the perception of overall social support (P = 0.02) as well as support from friends (P = 0.01) and significant others (P = 0.05) in the sildenafil-treated patients, while it remained unchanged in the control group. However, ESs for these measures were low, 0.31 for overall support, 0.24 for friends’ and 0.39 for significant others’ support, did not reflecting clinical relevance.

### 3.6. Adverse Effects

Sildenafil was well tolerated. The most common side-effect was headache, reported by 32% of sildenafil-treated patients (*n =* 16). Only one patient reported dyspepsia (2%). All of these adverse effects were transient and mild in nature. No serious adverse events related to the study drug were reported.

## 4. Discussion

In our previous uncontrolled, open-label report [37] we indicated that sildenafil as an adjunct drug to standard treatment for men with alcoholism improved sexual function and overall satisfaction with intercourse in a clinically sound manner, as well as satisfaction with all specific domains of quality of life. The present open-label, controlled, multicenter study extends those preliminary findings showing that sildenafil treatment also significantly increased psychosocial outcomes of these patients such as wellbeing and functioning, self-esteem and the perception of social support as compared to a treatment as usual educational control group.

The magnitude of improvements observed in this trial was comparable to that observed in other clinical trials of sildenafil treatment for erectile dysfunction in depressive disorder, schizophrenia and PTSD [24,25,27] as well as in medical diseases and treatments [8,11,17,28–30,51]. Consistent with previous studies, the efficacy of sildenafil treatment in this study was statistically significant and clinically relevant across all the IIEF domains, under condition that effect sizes were interpreted according to Cohen’s classic demarcation of 0.2, 0.5 and 0.8 referring to small, moderate and large ES’s [48.^49^]. However, if ES equal 0.5 SD was taken into consideration as a universal measure of clinical significance [50], only changes in the IIEF domains concerning sexual desire and overall satisfaction could be interpreted as clinically relevant. Regarding potential impact of between-group differences in IIEF scores at baseline on outcome scores at endpoint, such effect was reliably neutralized in effect size calculation using the denominator that the pooled baseline values of both groups.

The effect of sildenafil on sexual functioning is not surprising, given that it is the primary target of its pharmacological action. This effect was noted in 98% of our patients, providing affirmative responses to global efficacy questions, concerning treatment-related improvement in erectile function and ability to perform sexual intercourse. What is surprising is the effects of sildenafil on virtually all the psychosocial outcomes.

ED is a complex condition, which depends upon various emotional, societal, and relationship factors [52,53]. A recent qualitative study [54] described the impact of ED on subjective feelings of 40 men, their expectations of sildenafil, and impact of treatment on themselves and their relationships. ED caused marked effects on self-esteem and their social relationships. Successful sildenafil treatment led to a significant improvement in wellbeing, confirming the beneficial effect on masculine self worth. In our previous study, we also showed that self-esteem was the primary mediating factor in the ED-quality of life relationship in 101 men suffering from AD and concomitant ED [55]. In line with data from several studies [53,56–58] we found that among those who responded to sildenafil there was a marked improvement not only in wellbeing and self-esteem, but also in general functioning, and perceived social support from friends and significant others.

In accord with these changes, we observed a significant reduction in symptoms of depression in the sildenafil group. Noteworthy, depressive symptoms reduced also among the control subjects. Recall here that patients with severe depression (BDI score >15) were excluded from the study. This restriction in range of depressive scores might alter the sensitivity of the measure of depression. The improvement in BDI scores in both groups was no related to a nonspecific effect of the repeated visits with the research team.

Following Rosen and colleagues’ study [59] conclusion, we can suggest that psychosocial changes associated with ED therapy may be mediated by changes in sexual function, mood, and family relationships and/or, as we observed, by improvements in the senses of self-esteem and social support outcomes. However, the precise mechanisms by which sildenafil converts the beneficial effects on ED into the psychosocial domains require further studies.

The main limitation of our study is its open-label nature and the lack of a placebo-control arm. It is unfortunate that no placebo group was used, since it is difficult to truly evaluate the clinical significance of change without the ability to compare it to placebo-induced change. Thus, a nonspecific, placebo effect with regard ED cannot be ruled out. However, such placebo effect seems unlikely, given the highly significant improvements in the multiple psychosocial and clinical variables achieved for a relatively short trial period. Moreover, these changes were not only statistically highly significant, but also appeared to be clinically relevant. Unlike a double-blind clinical trial, the open-label design is open for a clinician’s subjective bias during data collection and evaluation of study parameters, usually in favor of the efficacy of either experimental compound over comparator or vice versa. However, this is true mostly in respect to clinical impressions and assessments of symptoms, but not self-administered questionnaires, where the clinician’s impact on the outcome measurement is practically excluded. A potential bias regarding qualification level of physicians is precluded because, to our knowledge, the clinicians of all participating centers were equivalently trained and had equal experience.

The significant correlation between improvements in sexual functioning and psychosocial and clinical measures, we found in this study, seems to be genuine, and not artificial, implying a potential overlap between the underlying constructs. Unfortunately, because of a relatively short trial period we were not able to examine the effects of sildenafil on drinking patterns. This issue should be addressed in further longitudinal study. Nonetheless, given the benefits of sildenafil for patients, physicians and other healthcare professionals some authors argue that, with the provision of proper assessments, sildenafil should be made available as an over-the-counter medication [60].

In summary, this open-labeled comparative evaluation demonstrates that sildenafil addition was more effective for improving ED than standard treatment for alcoholism alone. There was little risk involved in sildenafil treatment, since potential adverse effects of sildenafil were limited only headache. The benefits are the confirmation of clinical efficacy and safe side-effect profile of sildenafil, as well as improvement of wellbeing, mood and social functioning of the patients with AD. The information obtained in the study is valuable for both clinicians and policymakers to develop innovative therapeutic strategies for treatment of ED in men with alcohol dependence.

## Figures and Tables

**Table 1.** Sociodemographic characteristics and alcohol history.

**Variable**	**Sildenafil group (n = 50)**	**Control group (n = 51)**	
Age (yr.)	44.0 (8.7)	43.5 (5.9)	t = 0.37, df = 99, P = 0.71
Marital status Single Married Divorced/separated/Widowed	6 (12) 31 (62) 13 (26)	2 (3.9) 41 (80.4) 8 (15.7)	χ^2^ = 4.57, df = 2, P = 0.12
Education (yr.)	11.1 (4.5)	10.2 (3.5)	t = 1.08, df = 99, P = 0.33
Employment status Employed Unemployed	16 (32.0) 34 (68.0)	16 (31.4) 35 (68.6)	χ^2^ = 0.005, df = 1, P = 1.0
Religious affiliation Jewish Non-Jewish	31 (62.0) 19 (38.0)	34 (66.7) 17 (33.3)	χ^2^ = 0.24, df = 1, P = 0.70
Immigration status Non-immigrant Immigrant	23 (46.0) 27 (54.0)	38 (74.5) 13 (25.5)	χ^2^ = 8.58, df = 1, P = 0.004
Length of immigration (yr.)	16.4 (13.3)	18.1 (15.4)	t = 0.42, df = 51, P = 0.68
Age at first alcohol consumption (yr.)	17.2 (3.4)	21.3 (7.0)	t = 3.75, df = 99, P < 0.001
Age at first binge (yr.)	20.5 (6.4)	26.1 (8.6)	t = 3.68, df = 99, P < 0.001
Duration of harmful alcohol consumption (yr.)	14.8 (9.7)	13.0 (10.4)	t = 0.91, df = 99, P = 0.36
No. of prior inpatient detoxification(s) 0 1 2+	30 (60.0) 13 (26.0) 7 (14.0)	28 (54.9) 14 (27.5) 9 (17.6)	χ^2^ = 0.35, df = 2, P = 0.81
Average alcohol intake in last 6 months (g alcohol/drinking day)	700 (648.8)	694 (453.8)	t = 0.048, df = 99, P = 0.96
No. of drinking days in last month	8.6 (10.8)	6.8 (10.3)	t = 0.85, df = 99, P = 0.43

Mean scores ± SD are shown, if other not indicated.

**Table 2.** Summary of results on the International Index of Erectile Function.

**Sexual function domain**	**Sildenafil group (n = 50)**				**Control group (n = 51)**				**Effect size**
	**Baseline**	**Endpoint**	**t-value**	**P-value**	**Baseline**	**Endpoint**	**t-value**	**P-value**	

Total score	39.9(16.9)	56.7 (12.9)	7.23	<0.001	53.7 (10.8)	50.9(12.3)	1.90	0.64	1.25
Erectile function	15.9 (7.6)	23.2 (5.6)	6.79	<0.001	21.8 (5.3)	20.8 (5.7)	1.46	0.15	1.17
Orgasmic function	6.1 (3.1)	8.2 (2.1)	5.64	<0.001	8.0 (1.8)	7.4 (2.2)	2.27	0.03	1.01
Sexual desire	6.0 (2.2)	7.3 (1.4)	5.11	<0.001	7.1 (1.5)	6.6 (1.4)	2.34	0.02	0.91
Intercourse satisfaction	7.1 (3.7)	10.3 (2.8)	5.51	<0.001	9.5 (2.4)	9.0 (2.5)	1.38	0.17	1.11
Overall satisfaction	4.7 (2.5)	7.6 (2.3)	8.35	<0.001	7.3 (1.9)	7.0 (2.0)	1.16	0.25	1.23

Paired t-tests, two-tailed.

**Table 3.** Summary of psychosocial outcomes.

**Outcome measure**	**Sildenafil group (n = 50)**				**Control group (n = 51)**				**Effect size**
	**Baseline**	**Changes from baseline to endpoint**	**t-value**	**P-value**	**Baseline**	**Changes from baseline to endpoint**	**t-value**	**P-value**	
General Life Functioning	3.5 (0.7)	−0.5 (0.6)	5.04	<0.001	3.5 (0.6)	−0.09(0.5)	1.41	0.17	0.63
Well-being	3.4 (0.7)	−0.4 (0.7)	4.72	<0.001	3.6 (0.7)	−0.009 (0.5)	1.25	0.22	0.44
Functioning	3.5 (0.7)	−0.5 (0.7)	4.57	<0.001	3.5 (0.7)	−0.1 (0.6)	1.18	0.24	0.57
Beck Depression Inventory	10.5 (6.2)	4.9 (5.0)	6.93	<0.001	9.6 (6.1)	2.0 (4.8)	2.98	0.01	0.47
General Self-Esteem Scale	15.2 (3.7)	−2.4 (3.9)	4.27	<0.001	15.5(3.6)	−0.2 (3.4)	0.46	0.65	0.61
MSPSS, total score	54.4 (8.9)	−3.2 (8.9)	2.52	0.02	51.7(14.2)	0.5 (9.3)	0.38	0.71	0.31
Family	17.8 (4.7)	−1.0 (4.4)	1.59	0.12	17.4 (6.0)	−0.2 (3.8)	0.30	0.77	0.15
Friends	16.8 (4.7)	−1.1 (3.0)	2.68	0.01	15.9 (5.9)	0.2 (3.4)	0.41	0.68	0.24
Significant others	19.8 (3.5)	−1.1 (3.8)	1.96	0.055	18.5 (4.6)	0.5 (3.5)	0.91	0.37	0.39

* - Paired t-rests, two-tailed

MSPSS, Multidimensional Scale of Perceived Social Support (45).

## Abbreviations:

ED: erectile dysfunction;
AD: alcohol dependence;
IIEF: International Index of Erectile Function;
PTSD: Post Traumatic Stress Disorder;
AASD: Alcohol-associated sexual dysfunction;
GLF: Global Life Functioning inventory;
BDI: Beck Depression Inventory;
RGSES: Rosenberg’s General Self-Esteem Scale:
MSPSS: Multidimensional Scale of Perceived Social Support;
SD: standard deviations;
ES: effect size.
